# Nucleic Acid Biomarkers in Waldenström Macroglobulinemia and IgM-MGUS: Current Insights and Clinical Relevance

**DOI:** 10.3390/diagnostics12040969

**Published:** 2022-04-12

**Authors:** Daniela Drandi, Philippe Decruyenaere, Martina Ferrante, Fritz Offner, Jo Vandesompele, Simone Ferrero

**Affiliations:** 1 Department of Molecular Biotechnology and Health Sciences, Hematology Division, University of Torino, 10126 Torino, Italy; martina.ferrante@unito.it (M.F.); simone.ferrero@unito.it (S.F.); 2 Department of Hematology, Ghent University Hospital, 9000 Ghent, Belgium; fritz.offner@ugent.be; 3 OncoRNALab, Cancer Research Institute Ghent (CRIG), 9000 Ghent, Belgium; jo.vandesompele@ugent.be; 4 Department of Biomolecular Medicine, Ghent University, 9000 Ghent, Belgium

**Keywords:** WM, IgM-MGUS, *MYD88*, *CXCR4*, miRNA, lncRNA, cfDNA, liquid biopsy

## Abstract

Waldenström Macroglobulinemia (WM) is an indolent lymphoplasmacytic lymphoma, characterized by the production of excess immunoglobulin M monoclonal protein. WM belongs to the spectrum of IgM gammopathies, ranging from asymptomatic IgM monoclonal gammopathy of undetermined significance (IgM-MGUS), through IgM-related disorders and asymptomatic WM to symptomatic WM. In recent years, its complex genomic and transcriptomic landscape has been extensively explored, hereby elucidating the biological mechanisms underlying disease onset, progression and therapy response. An increasing number of mutations, cytogenetic abnormalities, and molecular signatures have been described that have diagnostic, phenotype defining or prognostic implications. Moreover, cell-free nucleic acid biomarkers are increasingly being investigated, benefiting the patient in a minimally invasive way. This review aims to provide an extensive overview of molecular biomarkers in WM and IgM-MGUS, considering current shortcomings, as well as potential future applications in a precision medicine approach.

## 1. Introduction

Molecular biomarkers are broadly used for diagnosis, treatment selection and disease monitoring in many clinical settings [1,2]. In the past decade, research in Waldenström macroglobulinemia (WM) has exemplified how nucleic acid analysis may lead to biomarker discovery, hereby enabling more accurate diagnosis and therapy selection. WM is a lymphoplasmacytic lymphoma (LPL) characterized by the predominant bone marrow (BM) accumulation of small lymphocytes, plasma cells (PC) and plasmacytoid lymphocytes. In WM, these abnormal cells are responsible for the overproduction of monoclonal immunoglobulin M (IgM) paraprotein. This abnormal proliferation of neoplastic B-cells impairs the BM equilibrium, hereby inducing cytopenias, and leads to an overabundance of monoclonal IgM, resulting in blood hyperviscosity [3,4]. From a pathological perspective, the WM heterogeneous cell population consists of different B-cells across a morphological continuum, suggesting that the disease may originate during B-cell differentiation after somatic hypermutation in the germinal center and prior to isotype class switching [5,6].

**Figure 1:**
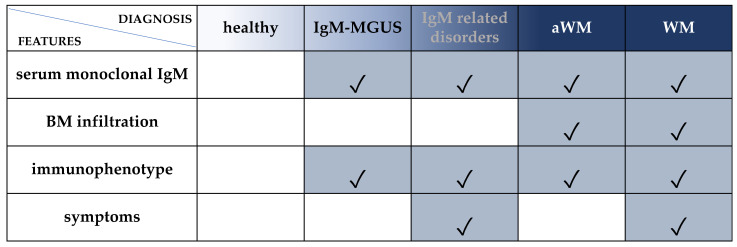
Diagnostic criteria for WM and IgM-MGUS. **Serum monoclonal IgM:** any concentration according to Owen 2003 (2° IWWM), Swerdlow 2008 (WHO 2008) and Campo 2011 (WHO 2011) [9,11,13] or ≥30 g/L according to Ansell 2010 (mSMART), Swerdlow 2017 and Maqbool 2020 [14,17,19]. **BM infiltration:** unequivocal BM infiltration by lymphoplasmacytic lymphoma [9,11] or infiltration ≥10% [14,17,19]. **Immunophenotype**: immunophenotype consistent with WM: CD19+, CD20+, CD22+, CD79+, CD5-, CD10-, CD23-. Of note, 5–10% of WM cases could express CD5 [20,21]. **Symptoms:** attributable to tumor infiltration (in BM or extramedullary) and/or to the monoclonal protein (related to immunological or chemical properties) [7,8,21]. **IgM-related disorders**: patients who have clinical features attributable to the IgM monoclonal protein but without overt evidence of LPL in the BM. For cases in which BM infiltration is not confirmed, the immunophenotypic profile is useful to discriminate the pattern of WM from other IgM-related disorders. However, BM infiltration by immunohistochemistry is mandatory for a definitive WM diagnosis. **IgM-MGUS**: patients with an IgM gammopathy, without evidence of LPL in the BM biopsy and no symptoms. Cases with detectable clonal B cells by immunophenotype and absence of BM infiltration by LPL [9] or BM infiltration <10% and IgM <30 g/L [14,17,19] should be classified as IgM-MGUS. **aWM:** patients with an IgM gammopathy and BM infiltration by LPL without symptoms. Immunophenotyping is strongly recommended for differential diagnosis. **WM**: patients with IgM protein of any concentration and unequivocal BM infiltration and symptoms. Immunophenotyping is strongly recommended.

WM belongs to the spectrum of IgM gammopathies, encompassing a wide and heterogeneous group of hematological conditions, ranging from asymptomatic IgM monoclonal gammopathies of undetermined significance (IgM-MGUS), through symptomatic IgM related disorders (such as IgM gammopathies of renal or neurological significance), to asymptomatic WM (aWM), and ultimately to symptomatic WM [7,8,9,10,11,12]. Therefore, an appropriate diagnostic classification that can distinguish between these different entities is crucial. The Second International Workshop Criteria (2° IWWM) formulated the following requirements for WM diagnosis: (a) the presence of IgM monoclonal gammopathy of any size, (b) a BM trephine biopsy with lymphoplasmacytic infiltration and (c) an immunophenotype that excludes the possibility of other lymphoproliferative disorders [9] (Figure 1). Of note, the different diagnostic criteria have been updated several times, and caution should be exercised when comparing between studies [11,13,14,15,16,17,18] (Figure 1).

Patients with IgM-MGUS can progress to WM at a rate of 1.5% to 2% per year [22]. Approximately 20–25% of WM patients are asymptomatic at the time of diagnosis, but up to 70% of these patients will eventually develop disease-associated symptoms within 10 years [23].

WM was described for the first time in 1944 and the role of genetic factors in the pathogenesis has been suggested early on, based on prior observations of familial aggregation [24,25]. Since then, a growing number of studies on the genomic and transcriptomic profiles of WM and IgM-MGUS have pointed out the complexity of these hematological diseases [10]. Although clearly associated with del(6q21) and del(13q14), the relatively low frequencies (40% and 10% respectively) of these aberrations and their detectability in other B-cell lymphoproliferative disorders (i.e., chronic lymphocytic leukemia (CLL) or multiple myeloma (MM)) did not allow for their straightforward use in WM diagnosis [11,26]. In 2012, the first whole-genome sequencing (WGS) study by Treon et al. revolutionized genetic insights by demonstrating a somatic mutation in myeloid differentiation primary response 88 gene (*MYD88^L265P^*) in 90% of WM and 10% of IgM-MGUS cases [27]. These findings were soon reproduced in larger patient series, and increasingly sensitive methods confirmed the presence of *MYD88^L265P^* in more than 95% of WM patients. Moreover, WGS identified several other highly prevalent somatic mutations in *CXCR4 (CXCR4^WHIM^* or *CXCR4^MUT^)* and *ARID1A* genes [27,28]. Of note, genetic factors have not yet been included in the current diagnostic and prognostic (International Prognostic Scoring System (IPSS)) criteria of WM [29,30]. Besides mutational profile, different classes of coding and non-coding RNA have also increasingly been investigated as these molecules may have crucial roles in disease onset and progression by regulating gene expression and cellular function, and may serve as potential biomarkers [31,32,33].

In recent years, liquid biopsy has been proposed as an alternative to invasive tissue biopsy [34,35,36]. Potential advantages include its minimally invasive nature, its ability to reflect spatial inter- and intra-tumor heterogeneity and the possibility to provide longitudinal monitoring through repeated sampling [37]. The development and use of circulating, reliable biomarkers through liquid biopsy sampling for the diagnosis, therapy response prediction and prognostication of WM could significantly impact daily clinical practice. However, critical challenges such as method standardization and sensitivity must be overcome to facilitate its translation from bench to bedside.

This review aims to present current knowledge regarding nucleic acid biomarkers in WM and IgM-MGUS to elucidate their role and highlight their translational potential as precision medicine biomarkers. Moreover, the biological mechanisms thought to be involved in IgM-MGUS to WM progression will be highlighted.

## 2. DNA Biomarkers

### 2.1. The Hallmark Genomic Alterations

#### 2.1.1. MYD88^L265P^ and CXCR4^MUT^

The most noteworthy finding in WM has been the discovery of two activating somatic mutations affecting the *MYD88* and *CXCR4* genes [27]. *MYD88^L265P^* is the most recurrent mutation in the genomic landscape of WM and is found in approximately 90% of the patients (Table 1).

MYD88 is an adaptor protein that acts downstream of the Toll-like and interleukin-1 receptors (TLR/IL1R), which are both implicated in the innate immune response through a similar signaling cascade [68]. A common characteristic of innate immune receptor signaling is the self-clustering of proteins into oligomeric complexes, known as supramolecular organizing centers (SMOCs) [69,70]. TLR/IL1R activation, through the homotypic TIR domain interactions, triggers the oligomerization of MYD88 and the assembly of a multifunctional organizing center, named MYDDosome [71]. The dynamics of protein recruitment and stepwise assembly of the MYDDosome in TLR/IL1R signal still need to be fully elucidated. Although numerous structural conformations are possible, it has been observed that the size of the MYD88 oligomers (>4 MYD88s) is a decisive factor in the IL1R signal transduction and is crucial for recruiting and binding other post-receptor signal transducers, such as interleukin-1 receptor-associated kinases (IRAK4 and IRAK1) or bruton tirosine kinase (BTK), resulting in sustained NF-κB signaling [71,72]. Notably, it has been shown that the *MYD88^L265P^* mutation, seated in the TIR domain, (and not the other no-L265P mutations) has an increased propensity to build extremely stable oligomers, compared to the wild-type protein, leading the MYDDosome formation and thereby constitutive NF-κB activation, contributing to cell proliferation, cytokine secretion (i.e., TNF, IL-6, IL-1) and malignant cell survival [50,73,74].

Another pro-survival signal supported by *MYD88^L265P^* involves the hematopoietic cell kinase (HCK), a member of the SRC tyrosine kinases family that, when activated by IL-6, triggers signaling through BTK, PI3K/AKT, and MAPK/ERK1/2 [75]. Lastly, WM cells can also transship *MYD88^L265P^* via extracellular vesicles (EV), which prompts inflammatory signaling in the absence of receptor activation and might strongly contribute to a growth-supportive proinflammatory microenvironment [76].

In clinical practice, *MYD88^L265P^* aids in supporting the diagnosis of WM and helps differentiate from other IgM-secreting lymphoid malignancies, such as marginal zone lymphoma (MZL) and IgM multiple myeloma (MM), where it is less frequently mutated or absent, respectively. Moreover, *MYD88^L265P^* is detected in more than 50% of IgM-MGUS patients, 10% of whom can evolve to WM and has been observed that those with a higher mutated allele burden (mutant allele relative to wild type) have a greater risk to progress to WM [40,77]. Of interest, *MYD88^L265P^* by itself does not seem to have primary oncogenic effects, as has recently been demonstrated in mouse models [78,79,80].

**Figure 2:**
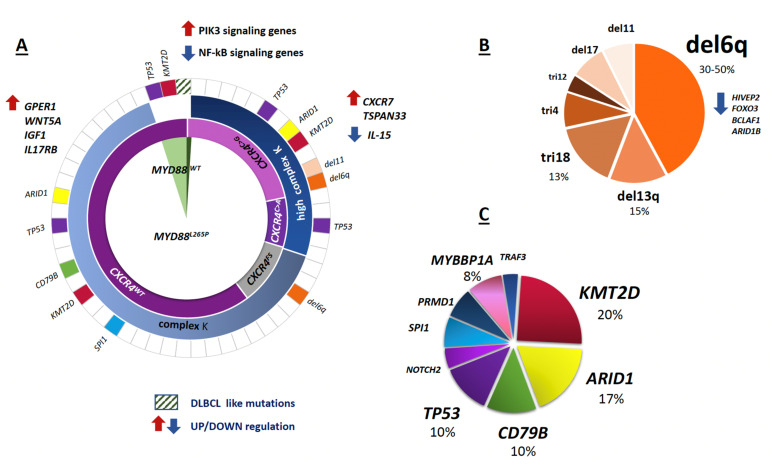
Mutational and cytogenetic landscape of WM. The figure describes the association between genomic abnormalities in WM patients. (**A**) From the center outward, the distribution and overlap of *MYD88^L265P^* mutations, *CXCR4^MUT^* mutations, karyotype (K) (Complex K: <5 clonal aberrations; high complex K: ≥5 clonal aberrations), copy number alterations (CNAs) and less frequent mutations (MUTs) are shown, respectively. The color code in the outer ring refers to colors in panels B and C. Relevant up and down-regulated genes (arrows) are reported. DLBCL like mutations: somatic mutations overlapping those detected in diffuse large B cell lymphoma (DLBCL), (i.e., *TBL1XR1*, *PTPN13*, *MALT1*, *BCL10*, *NFKB2*, *NFKBIB*, *NFKBIZ*, and *UDRL1F*). (**B**) Relative distribution of cytogenetic abnormalities. tri: trisomy, del: deletion. (**C**) Relative distribution of uncommon mutations. The percentage (%) of the most frequent MUTs and CNAs are estimated based on published data, for more details see below.

Despite having a similar histologic and transcriptional profile, *MYD88^L265P^* and *MYD88^WT^* patients exhibit distinct clinical features and an idiosyncratic genomic profile [81,82]. Indeed, copy number alterations (CNA) are common in *MYD88^L265P^*, as well as the prevalence of other somatic mutations, such as *CXCR4^MUT^* [10,82,83]. In *MYD88^WT^* patients, on the contrary, del(6q) is rare; *CXCR4* is usually wildtype, and the genomic profile is characterized by somatic mutations that overlap with those detected in DLBCL, such as *TBL1XR1, PTPN13, MALT1, BCL10, NFKB2, NFKBIB, NFKBIZ* and *UDRL1F* (downstream of BTK and IRAK) (Figure 2A) [82].

The second most common somatic mutation, observed in up to 40% of WM patients, occurs in the *CXCR4* gene (Table 2) [28,51,84,85]. CXCR4 is a chemokine receptor and member of the 7-transmembrane receptors family, that activates intracellular signaling pathways by binding to heterotrimeric G-proteins through its C-terminus segment [86].

More than 40 non-sense (NS) or frameshift (FS) mutations have been observed in the *CXCR4* gene (*CXCR4^MUT^*) [28,87]. The most common variant, representing over 50% of *CXCR4* mutations, is a non-sense C > A or C > G transversion in a highly conserved region at nucleotide position 1013, responsible for the generation of a stop codon (S338X), resulting in the loss of 15 amino acids at the C-terminal region of the CXCR4 protein [88,89]. These *CXCR4^S338X^* nonsense mutations affect the expression and activity of CXCR4 mainly through the PI3K-AKT-NF-κB and the MEK1/2 and ERK 1/2 pathways, involved in cell proliferation, migration, and survival [90,91].

Practically all *CXCR4^MUT^* patients harbor *MYD88^L265P^*, suggesting the subclonal nature of *CXCR4^MUT^* with respect to *MYD88^L265P^* acquisition, and only rare cases of *CXCR4^MUT^/MYD88^WT^* have been reported (Figure 2A) [28,60,87,93,94]. Moreover, *CXCR4^MUT^* shows a highly variable clonal distribution in WM and IgM-MGUS patients and particularly *CXCR4^S338X^*, as opposed to *CXCR4^FS^* mutations, are associated with complex karyotypes [84,87].

In a recent case study in a *CXCR*4^MUT^ patient, WGS highlighted alterations in genes associated with DNA damage repair (DDR) (*UVRAG* gene), tumor suppression (*BTG220, DAB2*), chromosome instability (*MACROD2, CCSER1*), cell cycle regulation (*SCAPER*) and post-translational protein modifications (*LNX1* and *DCUN1D4*). However, further analysis of 46 WM patients did not show a significantly different distribution of these mutations between *CXCR4^WT^* vs. *CXCR4^MUT^* patients [95].

*MYD88^L265P^* and *CXCR4^MUT^* were initially assessed on CD19+ (CD19-selected cells) BM samples using allele-specific quantitative polymerase chain reaction (AS-qPCR) and Sanger sequencing. Subsequently, many studies have used unselected BM samples and distinct assays and methods with different levels of sensitivity (Table 1 and Table 2). Both mutations can be detected not only in BM, PB (of note, B-cell–depleting agents, particularly rituximab, can greatly decrease mutation detection rate in PB) and plasma but also in skin, cerebrospinal fluid (CSF) and pleural effusions [62,96,97,98].

As of today, a gold standard molecular method for *MYD88* and *CXCR4* mutation detection is lacking. Although CD19+ cell sorting can improve the mutation detection sensitivity, cell selection is not cost-effective and not applicable to all clinical laboratories [10,27,38,40,43,47,49,60,97]. A recent study demonstrated that in unselected BM samples, AS-qPCR was superior in detecting *CXCR*4^S338X^ compared to amplicon massively parallel sequencing (MPS) (63% vs. 16%) [92]. Additionally, sensitivity of both methods was higher for *MYD88^L265P^* detection (98% and 69% respectively), confirming the subclonal nature of *CXCR4^S338X^* but also indicating a direct dependence of MPS performance on the level of BM involvement [58,67,92].

Consistently, a strong correlation between the mutational burden and the number of pathological cells has been demonstrated in unsorted material [40,42]. Therefore, the *MYD88^L265P^*/*MYD88^WT^* ratio might also be proposed as a quantitative marker and useful diagnostic tool for MRD analysis. Recently, digital PCR (dPCR) has been described as more sensitive than AS-qPCR across different specimen types (including plasma-cfDNA), for *MYD88^L265P^* screening and MRD analysis, suggesting that the implementation of dPCR assay in routine diagnostic laboratories might avoid the need for CD19+ selection [62,99].

#### 2.1.2. 6q21 Deletion

Small CNAs involving B-cell regulatory genes are highly prevalent in WM [28]. Paiva et al. showed that the frequency of patients displaying CNAs significantly increased with disease stage (IgM-MGUS (36%), aWM (73%) and WM (82%)) [100].

The 6q21 deletion (del(6q)) is the most frequent cytogenetic aberration and is detected by fluorescence in situ hybridization (FISH) in up to 30–50% of WM cases [83,101,102,103,104]. Other less frequent cytogenetics abnormalities include del13q (15%), trisomy 18 (10%), trisomy 4 and del17p (8%) (Figure 2B) [26,105,106]. Chromosome 6q deletion, mostly from q14 to q27, contains negative regulators of the MYD88/NFκB pathway (BLIMP1, TNFAIP3, HIVEP2, TRAF3IP2, IRAK1BP1), BTK inhibitors (IBTK) as well as controllers of apoptosis and differentiation (FOXO3, BCLAF1, PERP) [21,83,107,108,109].

So far, limited and discordant data linking molecular and cytogenetic information are available. Despite the initial observation that del(6q) and *CXCR4* mutations are mutually exclusive, conflicting data have been reported [83,84,103]. In a cohort of 219 patients, Krzisch et al. found that 35% of del(6q) cases harbored *CXCR4* mutations, as detected by chromosomal banding analysis (CBA), FISH, and targeted MPS. Moreover, a significantly more complex karyotype was shown in patients with del(6q) [110].

Cytogenetic studies may be useful to detect del(6q) as well as other abnormalities that might aid in differential diagnosis and outcome prediction [26]. However, the difficulty to obtain tumor metaphases in vitro due to the low mitotic index of the tumor cells and the need for CD19+ BM cells selection hampers the employment of CAB and FISH analysis for routine diagnostic assessment in WM patients.

### 2.2. Infrequent DNA Mutations

Albeit at low frequency, other recurring somatic mutations have been reported, including *ARID1A* (17%), *CD79B*, *KMT2D* (or *MLL2*), *MYBBP1A* and *TP53* (<15% of cases each) (Table 3, Figure 2C) [26,27,28].

Using MPS in a large series of WM and IgM-MGUS patients, Varettoni et al. (2017) demonstrated subclonal mutations in *KMT2D* (16%), *TP53* (8%), *NOTCH2* (7%), *PRDM1* (4%), *ARID1A* (3%), *CD79B* (3%) and *TRAF3* (1%), while no mutations were found in *MYBBP1A* and *TNFAIP3* [10]. Of note, the median number of *KMT2D* mutations was significantly higher in WM compared to IgM-MGUS patients. A subsequent study evaluating the 12 most frequently mutated genes confirmed an increased mutational load in different stages of monoclonal gammopathies: 21% in IgM-MGUS (additional mutations in at least 1/12 genes), 35% in aWM (by 8/12 genes) and 50% in symptomatic WM (by 12/12 genes) [93]. A recent study by Roos-Wiel et al. identified a novel recurring activating somatic mutation (p.Q226E) in the hematopoietic transcription factor SPI1 in 6% of patients, leading to altered gene expression programs responsible for oncogenic proliferative signaling and for blocking B cell differentiation [112]. This finding has been supported in a larger series of WM patients [110].

### 2.3. Impact of Somatic Mutations on Outcome and Therapy Response

It has been widely demonstrated that *MYD88* and *CXCR4* mutations have both diagnostic and therapeutic implications in WM. So far, four distinct subsets of WM patients with peculiar clinical features, different outcomes and drug responses have been identified: *MYD88^L265P^*/*CXCR4^MUT^*, *MYD88^L265P^*/*CXCR4^WT^*, *MYD88^WT^*/*CXCR4^WT^* and *MYD88^WT^*/*CXCR4^MUT^*.

*MYD88^L265P^*/*CXCR4^S388X^* patients show a higher BM disease burden, higher serum IgM levels and are more likely to have symptomatic disease compared to *MYD88^L265P^*/*CXCR4^FS-MUT^*, *MYD88^L265P^*/*CXCR4^WT^* or *MYD88^WT^*/*CXCR4^WT^* patients that show respectively a lower and the lowest (*WT/WT*) BM disease involvement. Discordances between studies in WM have been reported, but the most supported observation is that *MYD88^L265P^*/*CXCR4^MUT^* patients show lower, later and less deep responses to BTK inhibitors (mainly ibrutinib), eventually resulting in shorter PFS compared to *MYD88^L265P^*/*CXCR4^WT^* patients [61,113]. *MYD88^WT^*/*CXCR4^WT^* cases, on the other hand, show resistance to targeted drugs (i.e., BTK-inhibitors, but also PI3K and mTOR inhibitors) and are characterized by an increased risk of disease transformation to high-grade lymphoma or of developing a therapy-related myelodysplastic syndrome (t-MDS), both leading to a poor OS [61,114,115]. Additionally, *MYD88^WT^* patients with DDR mutations represent a subgroup with ultra-high-risk disease [82,114]. Lastly, outcome and therapy response are still a challenge to face in the small subgroup of *MYD88^WT^*/*CXCR4^MUT^* patients [116].

Recently, multicenter phase II and III trials comparing the efficacy and safety of novel BTK inhibitors to ibrutinib, hinted at a higher efficacy of acalabrutinib and zanubrutinib in a small subset of *MYD88^WT^* patients, by showing overall and major response (at least a partial response [PR]) rates comparable to *MYD88^L265P^* cases [117,118,119]. However, both the heterogeneity of the methods adopted for mutation detection (from Sanger to targeted MPS), and the low limit of detection (0.5%), could have led to a misclassification of patients with a mutation level below the sensitivity of the employed methods [119]. Therefore, additional, more standardized studies, as well as longer follow-up cohorts are needed to better clarify the real impact of novel BTK-inhibitors in molecularly-driven subgroups of WM patients.

Within the uncommon mutations, *ARID1A* mutations are associated with greater tumor involvement [115]. Mutations in *CD79B* are mainly observed in *MYD88^L265P^*/*CXCR4^MUT^* patients, with the exception of two studies in which the co-expression of *CD79B* and *MYD88^L265P^*/*CXCR4^WT^* was associated with disease transformation and progression [84,120]. Despite being rare, trisomy 4, *SPI1* and *TP53* mutations have been associated with aggressive disease course and shorter OS. Data concerning the coexistence of *TP53* mutations with both *MYD88^L265P^* and *CXCR4^MUT^* and the activity of ibrutinib in this group of patients are conflicting [110,111,121]. The few available studies regarding the impact of cytogenetic abnormalities in WM reported shorter progression-free survival (PFS) in del17p patients, as well as more symptomatic disease, shorter time to treatment and poorer clinical outcomes (both PFS and OS) in del6q cases [26,83,100,103,105,110].

## 3. RNA Biomarkers

Approximately 80% of the human genome is transcribed into RNA, of which only 1.5% is protein-coding mRNA, with the rest being termed non-coding RNA (ncRNA). Current classifications differentiate between short ncRNAs (less than 200 nucleotides, including miRNAs) and large ncRNAs (larger than 200 bases, generally termed lncRNAs, but also including circRNAs) [122]. RNA molecules have unique properties that make them attractive potential biomarkers. Since these molecules mediate or influence intercellular communication, they may lead to an improved understanding of differentially expressed key pathways involved in lymphoma initiation and transformation. Moreover, the dynamics of RNA patterns may reflect functional, longitudinal changes in both the tumor and the non-malignant compartment during disease course or treatment.

### 3.1. Coding mRNA

Gene expression profiling studies showed that lymphoplasmacytic WM cells (CD138+/CD19+) have a homogeneous transcription profile with an mRNA signature that resembles CLL and normal B cells but clearly differs from MM and normal plasma cells (NPC). Both WM and CLL are indolent lymphomas that are likely to be derived from memory B-cells, which might explain the similar B-cell-like signature [123,124]. Among the 73 genes differentially expressed in WM compared to CLL and MM, IL-6 was most significantly upregulated. Increased IL-6 mRNA and protein levels have been reported in other studies and are thought to promote IgM secretion and WM cell growth [125,126,127,128]. Gene ontology analysis based on WM unique genes showed activation of the MAPK pathway, which is also involved in IL-6 signaling [124]. By targeting IL-6 with tocilizumab, a reduction in tumor growth rate and IgM secretion has been demonstrated in vivo [129]. These results support its role in the WM tumor microenvironment (TME) and its potential as a therapeutic target.

As the WM clone is comprised of B lineage cells ranging from B lymphocytes (BL) to plasma cells (PC), several studies have compared separate expression profiles of clonal CD19+ WM B-cells (WM-BL) and CD138+ WM plasma cells (WM-PC) with their respective normal counterparts (NBL and NPC, respectively). Using MPS, Hunter et al. presented the first transcriptional landscape of WM-BL compared to NBL. Upregulated genes included RAG1, RAG2, DNTT and IGLL1, involved in VDJ recombination, BCR signaling and somatic mutation. The class switch recombination gene AICDA was not observed, which is consistent with the lack of immunoglobulin class switching. Furthermore, upregulation of *CXCR4* and its ligand CXCL12 have been reported to increase cell adhesion to VCAM1, which might explain the homotypic cell clustering in WM patients [104]. Gutiérrez et al. identified a total of 171 and 498 genes that were differentially expressed between WM-BL and WM-PC compared to NBL and NPC, respectively. Further analysis illustrated the aberrant differentiation of clonal BL into PC by identifying 37 genes, including PAX5, whose expression level in WM-PC was intermediate between WM-BL on the one hand and MM-PC/NPC on the other hand. CD79, BLNK and SYK, all targets of PAX5 and characteristic markers of B lymphoid cell identity, were upregulated in WM-PC, with an expression level more similar to WM-BL. BLIMP1 and IRF4 levels, which play crucial roles in PC differentiation, were decreased in WM-PC compared to MM-PC/NPC. These data suggest that lower PAX5 repression in WM-PC attributes to its phenotypic pattern of intermediate features between clonal BL and PC. This is in line with the finding that most genes that were exclusively dysregulated in WM-PC compared to MM-PC and NPC were also overexpressed in WM-BL, further suggesting that WM-PC results from an incomplete maturation process of clonal BL [128]. Of interest, another study could not identify a higher similarity between WM cells and normal memory B-cells compared to peripheral B-cells [104]. Lastly, Gaudette et al. showed that dysregulation of BCL-2 family members could help in discriminating between B-cell-like phenotype (WM-BL, NBL and CLL) and PC phenotype (WM-PC, NPC and MM), illustrating that its expression may be driven by the state of differentiation. WM-PC cells expressed BCL-2 family proteins at levels more similar to NPC than MM, which is indicative of a higher apoptotic threshold in WM cells [130]. Increased expression of the antiapoptotic gene BCL-2, as well as a decreased level of proapoptotic BAX, have been previously reported [104].

Regarding the impact of recurrent genetic alterations on transcriptional regulation, a study showed that expression profiles in *MYD88^WT^* patients were relatively heterogeneous with downregulation of NF-κB signaling-associated genes and upregulation of PIK3 signaling. Of the 1155 genes that were differentially expressed between *MYD88^L265P^* and *MYD88^WT^* patients, only 603 were identified in the *MYD88^L265P^*/*CXCR4^WT^* vs. *MYD88^L265P^*/*CXCR4^WHIM^* signature. As *CXCR4^WHIM^* mutations are found almost exclusively in *MYD88^L265P^* patients, *CXCR4^WHIM^* mutations appear to counteract tumor suppressor upregulation in response to mutant *MYD88^L265P^* signaling, as evidenced by the normalization for TLR4 signaling associated gene expression and upregulation of *IRAK3*. In the *MYD88^L265P^*/*CXCR4^WT^* genotype, a marked increase of GPER1, WNT5A, IGF1 and IL17RB expression was shown, in which IL17RB and IGF1 activate NF-κB and AKT1/MAPK signaling, respectively [104,131]. The strongest gene markers for *MYD88^L265P^*/*CXCR4^WHIM^* patients were the upregulation of *CXCR7* and *TSPAN33*, as well as suppression of *IL-15* [104]. Concerning 6q deletions, Chng et al. found no differentially expressed genes between deleted and non-deleted cases [124]. This is in contrast with the study of Hunter et al. that included a larger number of WM samples, and demonstrated that 6q deletions were associated with over 131 differentially expressed genes, including suppression of the *NF-κB* negative regulator *HIVEP2*, as well as *BCLAF1, FOXO3* and *ARID1B* [104].

Regarding their potential use as biomarkers in WM, a study has shown that the level of mRNA expression was greatly dependent on the extent of BM involvement, with the expression profile of cases with low infiltration, clustering with normal plasmacytes [124]. In the same vein, one study showed that many genes relevant to WM biology, including *CXCL13, TP53, CXCR4, MYD88, CDC23* and *AKAP1* were significantly associated with BM disease involvement [104] (Table 4).

### 3.2. Non-Coding RNA

#### 3.2.1. miRNA

MicroRNAs (miRNAs) are short non-coding RNAs of ~22 nucleotides that play essential roles in almost all biological pathways, negatively regulating gene expression by targeting mRNA, typically at the 3′-untranslated region. Since miRNAs can target up to several hundred mRNAs, aberrant expression can influence a multitude of cell signaling pathways, including cancer onset and progression [31]. Several papers have investigated the role of miRNAs in WM/IgM-MGUS and their potential use as biomarkers (Table 5).

#### 3.2.2. Diagnostic Markers

Bouyssou et al. have shown that a 12-miRNA signature from extracellular vesicles was able to discriminate asymptomatic WM patients from healthy controls [138]. Hunter et al. identified 10 miRNAs that were differentially expressed in WM, of which five target the *IRS-PI3K* signaling pathway that plays a role in the growth and survival of WM cells: miR-29c (*PIK3R1*); miR-155 (*SHIP1*); miR-21 (*PTEN, PDCD4*); miR-145 (*IRS1*); and miR-126-3p (*IRS1, PIK3R2*) [132]. The combination of increased miR-320a and miR-320b levels as well as decreased miR-151-5p and let-7a levels has also been shown to distinguish WM from healthy controls, with the latter acting as a tumor suppressor by regulating different oncogenes such as *MYC* [140,142]. Fulciniti et al. have shown a decreased miR-23b expression in WM and identified an MYC/miR-23b/SP1 feed-forward loop, in which *c-MYC* acts jointly with *SP1* to downregulate miR-23b expression [136]. Gain of function studies showed a decrease in cell proliferation and lower colony formation ability, indicating a tumor suppressor role by reducing SP1-driven NF-κB activity. Treatment with IL-6 or supernatant from BM stromal cells resulted in a further decrease in miR-23b levels, illustrating the role of the human bone marrow TME in its expression [136].

**Figure 3:**
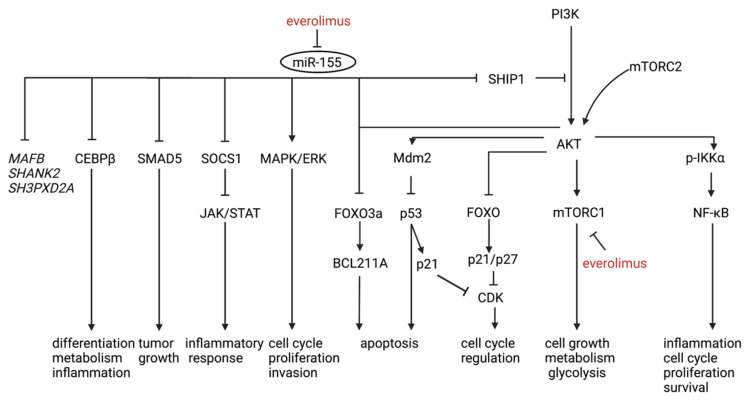
An illustrative overview of the pathways involved with increased miR-155 expression in WM. MAFB, SHANK2 and SH3PXD2A (italic) are more recently discovered targets of miR-155 in WM and further studies are needed to elucidate their role. Everolimus-dependent anti-WM activity is partially driven by targeting miR-155 (red).

Roccaro et al. identified a WM-specific miRNA signature characterized by increased expression of miR-363-5p/-206/-494/-155/-184/-542-3p, and decreased expression of miR-9-3p [135]. Decreased miR-9-3p and increased miRNA-155 levels have been associated with WM in other studies [132,137,139]. MiR-9-3p acts as a tumor suppressor by targeting protein kinases, oncogenes and transcription factors, thereby enhancing apoptosis as well as inhibiting B-cell differentiation and proliferation. Members of the miR-9 families are known to downregulate *PRDM1*, a significant regulator of B-cell development [139]. MiR-155 is also involved in essential pathways in different B-cell malignancies, including WM, targeting both AKT and NF-κB signaling pathways [131,143]. In two studies performing miR-155 LNA knockdown in WM cells, the role of miRNA-155 in WM proliferation and growth was confirmed in vitro and in vivo by targeting critical signaling cascades such as MAPK/ERK, PI3/AKT and NF-κB, which are involved in cell-cycle progression, adhesion, and migration [135,144]. Interestingly, stromal cells from miR-155-knockout mice led to significant inhibition of tumor growth, which suggests a role of miR-155 in WM proliferation both in the tumor and in the TME. Gene expression profiling revealed three known miR-155 targets (*SMAD5*, *SOCS1* and *CEBPβ*) as well as three novel targets (*MAFB*, *SHANK2*, and *SH3PXD2A*) [144]. Gaudette et al. observed a decreased *FOXO3* transcription factor and pro-apoptotic *BCL2L11* in cells with augmented miR-155-5p expression, hereby blocking apoptosis. Furthermore, mitochondrial priming can be induced by antagonism of miR-155, lowering the apoptosis threshold [130]. Lastly, miR-155 regulates proliferation through the cell-cycle transition. In miR-155 knockdown WM cells, G1 to S phase transition was blocked and associated with elevated transcripts for p53, p63 and p73, potentially providing a crucial alternate mechanism of cell growth arrest in the absence of p53. [135]. Figure 3 shows an illustrative overview of the pathways involved with increased miR-155 expression in WM.

Hodge et al. investigated specific miRNA signatures of different WM cellular subgroups. The miRNA signature of WM-BL consisted of mostly downregulated miRNAs compared to CLL and non-malignant B-cells, including miR-151, miR-335 and miR-342, whereas miR-373 was clearly increased in WM-BL. Most WM-PC clustered with MM-PC, yet retained a distinct miRNA profile of their own, characterized by the increased expression of more than 40 candidate miRNAs. No differentially expressed miRNA was detected between WM-BL and WM-PC, and no clear signature for lymphoplasmacytic (CD19+/CD138+) WM cells could be identified, possibly due to the clone’s morphologic diversity, sharing features of both BL and PC. After combining WM-BL, WM-PC and lymphoplasmacytic cells to mimic the heterogeneity observed in WM tumors, six miRNAs were differentially expressed in WM compared to nonmalignant B lineage cells (decreased expression of miR-152, miR-182, miR-373-5p and miR-575, with the opposite pattern for miR-21 and miR-142-3p) [139].

#### 3.2.3. Therapy Response and Prognostic Marker

Increased expression levels of miR-192-5p, miR-21-5p and miR-320b have been associated with disease progression, while expression of let-7d decreased with disease stage [138]. Increased expression of another 6-miRNA-signature (miR363-5p/206/494/155/184/542-3p) was associated with worse prognosis, predicted by the IPSS [135,145]. Treatment with rituximab, perifosine and bortezomib affected the expressions of the identified miRNAs (except miRNA-206), indicating the role of these miRNAs as therapy response predictors and possible targets for treatment [135]. Caivano et al. showed a trend for a positive association between a high EV miR-155 level and an intermediate-high IPSS score. More data are, however, needed to confirm these results [137].

Roccaro et al. illustrated ex vivo that everolimus targeted mTOR downstream signaling pathways in responders. Furthermore, everolimus induced toxicity, supported by cell-cycle arrest and caspase-dependent and -independent induction of apoptosis, even in the context of BM milieu, affecting migration, adhesion and angiogenesis. Through miR-155 loss-of-function studies, everolimus-dependent anti-WM activity was shown to be partially driven by targeting miR-155 [141]. It has been well documented that miR-155 targets *SHIP1*, which acts as a negative regulator of the PI3K/AKT and mTOR pathway [146]. Moreover, everolimus synergized with bortezomib and rituximab in targeting WM cells, as shown by synergistic inhibition of NF-κB/p65 activity and p-S6R, respectively—the latter through enhanced antibody-dependent cellular cytotoxicity. These results may support a rationale for combining everolimus with bortezomib or rituximab in certain patients that are refractory to everolimus monotherapy, as well as the use of miR-155 as a biomarker for therapy response prediction [141] (Table 5).

#### 3.2.4. miRNAs and Epigenetic Regulation

Besides genomic losses, epigenetic alternation represents the major mechanism by which gene expression is regulated and includes DNA methylation, histone acetylation and miRNA regulation [147]. A subgroup of miRNAs, called epi-miRNAs, actively modulate epigenetic processes via targeting mRNAs encoding methylating and acetylating enzymes. Histone acetylation is commonly deregulated through alterations in the balance between histone acetyltransferase (HAT) anddeacetylase (HDAC) activity, leading to enhanced gene transcription. Its role has been illustrated in various solid and hematological malignancies [148,149,150].

Roccaro et al. demonstrated that reduced expression of miR-9-3p and increased expression of miR-206-3p resulted in an unbalanced expression of HATs and HDACs at mRNA level in WM-BL, suggesting that histone modification plays a role in the pathogenesis. Specifically, miR-206-3p was found to target HAT KAT6A and miR-9-3p to target HDAC4 and HDAC5. Restoring miR-9-3p levels resulted in induction of toxicity in WM cells, supported by downmodulation of HDAC4 and HDAC5 and upregulation of acetylhistone-H3 and -H4, which led to induction of apoptosis and autophagy [133]. As mentioned, increased expression levels of miR-15a-5p and miR-16-5p have been found in WM patients [138]. In CLL, it has been shown that HDACs overexpression mediates the epigenetic silencing of miR-15a and miR-16. HDAC inhibition-induced expression of miR-15a and miR-16 was associated with decreased Mcl-1 levels, mitochondrial dysfunction and induction of cell death in CLL cells [151]. Furthermore, miR-15a has also been shown to target p53 in a miRNA/p53 feedback circuitry [152]. In MM, microRNA-15a/-16 regulates proliferation and growth of MM cells in vitro and in vivo by inhibiting AKT serine/threonine-protein-kinase (AKT3), ribosomal-protein-S6, MAP-kinases and NF-κB-activator MAP3KIP3 [153]. Del(13q14), which includes the miR-15a-5p/16-5p locus, has been described in 10% of WM patients [3]. Future research could focus on the role of these (epi)miRNAs and their interaction with dysregulated histone acetylation in WM.

In recent years, there has been increased interest in targeting epigenetic modulators by small molecule inhibitors. Different HDAC inhibitors (SAHA, TSA, panobinostat (LBH-589), and sirtinol) demonstrated dose-dependent killing and had at least additive antitumor effects when combined with bortezomib in a WM cell line [134]. In a phase two trial of panobinostat in relapsed/refractory WM patients, partial remission and minimal response were seen in 22% and 25% of cases, respectively. In addition, 50% of patients achieved stable disease and none showed progression while on therapy [154]. BET inhibitors are another class of molecules that inhibit cell proliferation by targeting bromodomain proteins (BRD2, BRD3, BRD4 and BRDT), resulting in decreased *MYC* expression through transcriptional regulation. A recent study that investigated two BET inhibitors (iBET and JQ1) in WM showed reduced cell proliferation in a dose-dependent manner. There was only a moderate effect on cell viability, which may be explained by increased anti-apoptotic BCL-2 expression, suggesting that targeting BCL-2 may be effective in inducing WM cell death. Indeed, combined treatment of JQ1 and venetoclax enhanced apoptosis. Notably, the efficacy was not compromised in the presence of the TME. Moreover, BET inhibitors were also shown to decrease stromal cell proliferation. This suggests that BET inhibition may influence the epigenetic regulation of both the tumor and the TME. When HDAC and BET inhibitors were combined, synergistic effects on cell death were shown, even with a very low dose of panobinostat (LBH589) [155].

#### 3.2.5. LncRNAs

Long non-coding RNAs (lncRNAs) are a large and heterogeneous class of non-coding transcripts, greater than 200 nucleotides in length. Based on their relative positions to protein-coding genes, lncRNAs are commonly classified as intergenic, intronic, sense overlapping or antisense overlapping [156]. Although their function is still largely unknown, lncRNAs play essential roles in cellular and physiological processes, such as chromatin remodeling, transcriptional regulation, and posttranscriptional modification [32]. Aberrant expression, mutations and SNPs in an increasing number of lncRNAs have been found to be involved in tumorigenesis and metastasis. Their tumor-specificity as well as their stability in circulating body fluids make them attractive potential biomarkers and therapy targets [157].

To date, lncRNA expression in WM has not yet been investigated. In CLL and MM, however, dysregulation of multiple lncRNAs has been shown. In CLL, DLEU2 [158], BM742401 [159] and lincRNA-p21 [160,161] act as tumor suppressors, respectively, by regulating *NF-κB* signaling and via induction by p53. BIC acts as an oncomiR progenitor by being a host of miR-155 [162] and MIAT by forming a regulatory loop with *OCT4* [163]. Other dysregulated lncRNAs with a currently unelucidated mode of action include ZNF667-AS1/lnc-AC004696.1-1, lnc-IRF2-3, and lnc-KIAA1755-4 [164]. In MM, iGAS5 [161,165], DLEU2 [158] and MEG3 [166] have been described as tumor suppressors, respectively, by regulating the mTOR pathway, being host of the miR-15a/16-1 cluster and regulating *p53* gene expression. *MALAT1* and *TUG1* have been reported to act as oncogenes by respectively regulating the bioavailability of *TGF-β* and affecting the expression of cell cycle regulatory genes by binding *PRC2* [161]. Dysregulation of Lnc-SENP5-4/NCBP2-AS2, lnc-CPSF2-2, lnc-LRRC47-1/TP73-AS1, lnc-ANGPTL1-3 and lnc-WHSC2-2 have also been shown in MM, although their function remains unclear [165]. One study reported lower levels of HOTAIR, an epigenetic regulator of chromatin and known oncogene in different solid tumors, in MM patients [161]. Further research, however, is needed to elucidate its role in MM. Lastly, increased lncRNA H19 levels were detected in the serum of (bortezomib resistant) MM patients, which was associated with the disease- and ISS stage. H19 sponges miR-29b-3p, hereby enhancing *MCL-1* transcriptional translation and inhibiting apoptosis [167,168]. Since MM and CLL are B-cell neoplasms closely related to WM, the aforementioned lncRNAs could be further investigated. For example, dissecting the role of the DLEU2/miR-15a/16-1 cluster, which is located on chromosome 13q14.3, a region known to be involved in CLL, MM and WM could produce valuable new insights.

#### 3.2.6. CircRNAs

Circular RNAs (circRNAs) are a more recently discovered subclass of large ncRNA, with covalently closed ends and lengths between ~100 to thousands of nucleotides. CircRNAs originate from a host gene and are formed through a backsplicing event, ligating the 3′ end of an exon to the 5′ end of the same or an upstream exon. Being highly evolutionary conserved, circRNAs may function as direct or indirect regulators of host gene expression at the transcriptional level, as sponges or decoys for miRNAs or RNA binding proteins, regulators of protein translation or under certain circumstances even as templates for translation. Altered expression has been shown in different solid and hematological cancers and increasing evidence shows that circRNAs can be used as reliable (cell-free) biomarkers, as they are present in different human body fluids, are highly tissue-specific and are more resistant to exonucleases due to their closed structure [33,169].

To date, differential expression of circRNAs has not been studied in WM. In CLL, circRPL15 has recently been shown to sponge miR-146b-3p, thereby increasing *RAF1* levels, activating *MAPK* signaling and promoting cell growth [170]. Upregulation of circCBFB activates the *Wnt/β-catenin* pathway by binding miR-607 and thereby derepressing production of *FZD3*, stimulating proliferation [171]. Moreover, both circRNAs have been associated with worse OS [170,171]. Circ_0132266 acts as a tumor suppressor by sponging miR-337-3p, resulting in increased levels of *PML*, a known regulator of gene expression and cell viability [172]. In MM, 619 unique circRNAs were identified in a MM cell line through RNA sequencing, including circSMARCA5, circRP11-255H23.2, circIKZF3, circCD11A (ITGAL), and circWHSC1 (MMSET) [173]. CircSMARCA5 has a tumor repressor role by binding to miR-767-5p, thereby inhibiting cell proliferation and promoting apoptosis. Increased expression is associated with a higher complete remission rate, as well as improved PFS and OS [174]. Circ_0000190 also acts as a tumor suppressor by sponging miR-767-5p, which in turn prevents the repression of its target *MAPK4*, slowing down progression. Upregulation of circ_000190 was associated with longer PFS and improved OS [175]. As the circRNA spectrum in WM is currently uncharted territory, elucidating their expression and function could produce valuable insights into the pathogenesis and identify potential disease-specific biomarkers.

## 4. IgM-MGUS to WM Progression

Patients with IgM-MGUS have a significantly lower number of mutations than patients with WM. The high prevalence of *MYD88^L265P^* in IgM-MGUS patients (50–80%) suggests that this somatic mutation is most likely an early oncogenic driver (Table 1). Meanwhile, the low prevalence of *CXCR4*, *KMT2D* and *TP53* mutations (<10%), which usually occur in a later stage, indicates that *MYD88^L265P^* by itself is insufficient to explain the malignant transformation from IgM-MGUS to WM [10]. Multistep genetic and/or microenvironment changes might lead to the progression of IgM-MGUS to WM. Although several clinical studies have identified biomarkers associated with a higher risk of IgM-MGUS to WM progression [176,177,178,179], no reliable molecular markers have been defined and the biological mechanisms driving the progression between these two entities are still unknown. Extensive transcriptome gene expression analysis using microarrays on CD19+ and CD138+ cells of WM and IgM-MGUS samples demonstrated that BLs and PCs harbor distinct molecular signatures [180]. A more recent study identified a common gene set signature that characterizes B-cells of WM and IgM-MGUS subjects, compared to healthy donors. This set of nine genes (*HIST1H1B, EZH2, CHECK1, LEF1, ADAM23, RASGRP3, ADRB2, PIK3AP1, CDHR3*) might highlight new candidate markers in IgM-MGUS responsible for the risk of progression to WM [181]. Furthermore, several studies have investigated the difference in miRNA expression signatures between IgM-MGUS, aWM and WM. Bouyssou et al. found no differential expression between aWM, WM and relapsed WM patients, suggesting that (exosomal) miRNA changes may occur in an early stage [138]. The assumption that a subset of IgM-MGUS can be regarded as the precursor state of WM is further supported by a study that showed a similar mRNA expression profile between an IgM-MGUS case and WM, suggesting a shared phenotype [124]. The combination of miR-320a and miR-320b was, however, able to distinguish WM from IgM-MGUS and IgM-MM, and decreased levels of miR-320a were significantly associated with *MYD88^L265P^*. As levels of miR-320a negatively correlated with lymphoplasmacytic cells infiltration in the BM, the expression profiles in samples with higher BM involvement were more similar to their malignant counterparts, whereas patients with less involvement had samples that tended to cluster with the respective non-malignant cells [140].

## 5. Liquid Biopsy

Liquid biopsy is the process of investigating tumor-derived cells, cell-free nucleic acids, metabolites, proteins or extracellular vesicles through biofluid sampling without the need for tissue biopsy. Biological sources for liquid biopsy include PB, urine, cerebrospinal fluid, saliva and pleural effusions, among other body fluids [182].

In the past decade, there have been major advances in the identification of diagnostic, phenotype-defining and prognostic biomarkers in lymphoproliferative diseases, which might complement current classification and prognostic tools, as well as guide therapy choice in a precision medicine approach [183,184]. In blood-derived liquid biopsies, circulating cell-free tumor DNA (ctDNA) and RNA (ctRNA), circulating tumor cells (CTCs), and extracellular vesicles are released into the PB, reflecting the tumor-specific genetic profile of the primary tissue biopsy, as reported by independent studies in hematological malignancies [184,185,186].

Potential advantages of liquid biopsy include its minimally invasive nature, its ability to reflect spatial inter- and intra-tumor heterogeneity and the possibility of longitudinal profiling. To date, however, their analysis has only been implemented as a therapy decision-maker in solid tumors [187,188].

**Figure 4:**
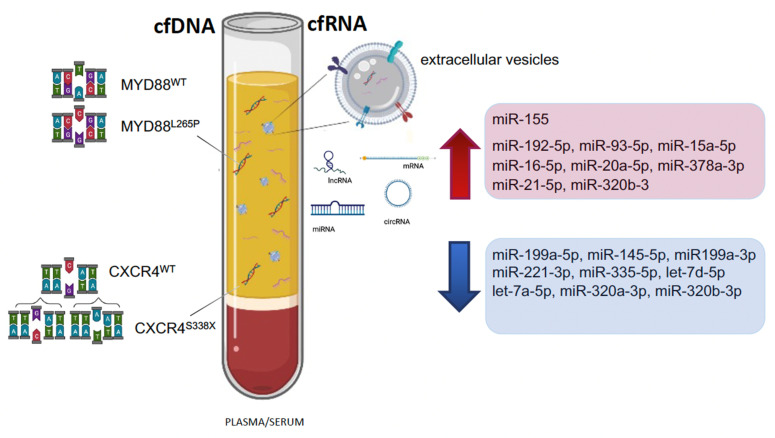
Cell-free DNA (cfDNA) and cell-free RNA (cfRNA) markers in plasma or serum of WM patients. Significant up- and downregulated miRNAs are shown (arrows).

In WM, ctDNA may represent a reliable “echo” of the tumor-specific genomic and epigenomic aberrations of the BM compartment, and even of extramedullary sites, and might be useful in assessing disease status, guiding therapeutic decisions and monitoring minimal residual disease (MRD) (Figure 4).

To this day, only a few studies have investigated the use of cfDNA in the characterization of the mutational landscape of patients with IgM monoclonal gammopathies.

A pioneering study by Bagratuni et al. compared the mutational status of *MYD88* and *CXCR4* in paired gDNA (from BM CD19+ selected cells) and cfDNA samples of patients with IgM monoclonal gammopathies. Qualitative allele-specific PCR and direct sequencing showed an overall concordance rate, between gDNA and cfDNA, of 94% and 90% for the most common *MYD88* and *CXCR4* mutations, respectively. These results were consistent among patient subgroups according to disease status (IgM MGUS, aWM, newly diagnosed WM, relapsed/refractory WM and WM in remission) [189]. A subsequent study by Wu et al., comparing BM and cfDNA data from WM patients for *MYD88^L265P^* and *CXCR4^S338X^* mutations by AS-qPCR, revealed only one discordance, related to an aWM patient with a slight BM infiltration [65]. Similarly, Demos et al. validated cfDNA for *MYD88^L265P^* and *CXCR4^S338X^* against CD19-selected and unselected BM and PB tissue fractions, strengthening the evidence that cfDNA can reliably be used to identify these two most common variants in WM patients [190]. Of note, the former studies have reported conflicting data regarding the correlations between BM infiltration, serum IgM levels and the concentrations of cfDNA [65,189,190]. Likewise, Drandi et al. demonstrated the feasibility of cfDNA analysis by dPCR in detecting *MYD88^L265P^* mutation in paired unselected BM and cfDNA samples from 60 WM patients. Their *MYD88^L265P^* dPCR assay showed an overall mutation detection rate on baseline unselected mononuclear cells samples of 95.3% in BM and 71.2% in PB. Interestingly, dPCR detected a log10 higher median *MYD88^L265P^* mutated/WT ratio in ctDNA compared to PB, while no statistically significant difference was observed between ctDNA and BM samples [62]. These data may contribute to the still open debate on whether to use sorted versus unsorted BM mononuclear cells to assess *MYD88^L265P^* mutation (Table 6).

Up to now, there is a lack of consensus regarding the optimal specimen and analytical method for mutational detection in WM, in terms of operating procedures, test sensitivity and result interpretation [99,192]. Researchers must be aware that differences in method sensitivity may lead to both a misclassification of disease status and an overestimation of the efficacy of novel treatments. In a recent publication, different PCR methods (qPCR vs. dPCR) have been compared in BM, PB, CD19+ and cfDNA samples: dPCR appeared to be the most sensitive approach for *MYD88* detection. Moreover, an algorithm was provided to suggest the most convenient PCR method based on available specimens and laboratory equipment [99]. Although highly relevant and promising, we are aware that the available data are premature to establish cfDNA as a single approach for disease screening and monitoring. Moreover, standardization of pre-analytical and analytical processes must be performed before integrating cfDNA analysis into the clinical practice. Currently, a multicenter clinical trial for non-invasive diagnostics and monitoring of MRD in WM and IgM-MGUS patients is ongoing (BIO-WM trial: NCT03521516), with the primary endpoint of demonstrating that the *MYD88^L265P^* mutation rate detected in cfDNA by dPCR is equivalent to the rate detected in BM.

Although most studies have focused on cfDNA, there has been an increased interest in different forms of circulating-free RNA biomarkers. Several mechanisms, such as encapsulation within extracellular vesicles (EVs) or ribonucleoprotein RNA-binding proteins complexes, protect circulating-free RNAs (cfRNA) from nuclease activity. Although the source and function of cfRNAs remain largely unknown, RNAs seem to be selectively packaged according to the viability and origin of the cells. Living cells seem to actively release RNA encased in large lipoprotein complexes, such as exosomes or microvesicles, while circulating RNA from dying cells is enclosed within apoptotic bodies or bound in protein complexes [193]. Besides RNA, EVs encapsulate different cellular components such as proteins, lipids, carbohydrates and DNA, thereby protecting them from degradation [194]. In different hematological malignancies, including WM, increased levels of EVs have been demonstrated. EVs have been shown to express malignancy-associated surface markers and to positively correlate with prognostic scores such as IPSS [195]. Furthermore, precursor miRNAs can be processed into mature miRNAs inside tumor-derived exosomes carrying the microRNA biogenesis machinery [196]. Recently, Mancek-Keber et al. highlighted an interesting role of EVs in lymphoma progression. Once EVs are internalized by the recipient cells, the transferred *MYD88^L265P^* recruits *MYD88^WT^* and triggers the activation of the NF-kB and the inflammatory pathway, both important for WM pathogenesis [76].

In WM, three studies have investigated circulating EV-derived miRNA expression and showed an upregulation of miR-192-5p, miR-93-5p, miR-15a-5p, miR-16-5p, miR-20a-5p, miR-378a-3p and miR-155 and a downregulation of miR-199a-5p, miR-145-5p, miR199a-3p, miR-221-3p, miR-335-5p, let-7d-5p, miR-320a-3p, miR-320b-3p, miR-151-5p and let-7a-5p, compared to healthy controls [137,138,140]. Moreover, increasing levels of miR-21-5p, miR-192-5p, miR-320b-3 and decreasing levels of let-7d-5p have been significantly associated with disease progression [138]. In the study of Kubizkova et al., both miR-320a and miR-320b were present in exosomes as well as in exosome-depleted samples. Interestingly, their levels tended to be enriched in exosomal fractions, which may indicate active transportation in EVs [140]. The study of Bouyssou et al. showed a lower correlation between miRNA expression in tumor cells and circulating exosomes derived from patient samples as compared to the correlation between miRNA levels of the cellular and exosomal fractions in WM cell lines. This may be explained by the presence of exosomes derived from various cell types including tumor cells, microenvironment cells and immune cells in the PB. Besides tumor-derived content, EV-derived miRNAs may therefore provide additional insights into changes in the TME and immune response during treatment [138]. The role of the TME is increasingly being recognized as a crucial factor in the pathogenesis of several B-cell malignancies, including WM, and to play a protective role in resistance to therapy [131,197,198]. Future research is needed to further elucidate the underlying mechanisms and ultimately enable translation in clinical practice (Table 7).

## 6. Conclusions

This review aimed to provide an overview of the molecular and cytogenetic landscape of WM and IgM-MGUS, as well as its clinical applications. In recent years, major progress has been made in genomic and transcriptomic profiling, hereby shedding light on the origin and evolution of the disease, unraveling involved pathways and dissecting the heterogeneity within the WM clone, as well as of the TME. Moreover, (cf)DNA and (cf)RNA biomarkers have been proposed that are able to define disease subgroups, are associated with disease progression and therapy response and allow minimally invasive detection of mutations.

An important limitation, however, is the discordancy between studies concerning diagnostic criteria, detection methods with ranging sensitivities and types of specimens analyzed (BM and PB, selected or unselected CD19+ B cells). Moreover, experimental details are often not reported, making data comparison challenging, especially in small patient cohorts such as the infrequent *MYD88^WT^*/*CXCR4^WT^* subgroup. Therefore, we encourage all authors to report sufficient experimental design details in order to allow a reliable comparison among studies. Likewise, there is a current lack of consensus between diagnostic laboratories on how to perform profiling analyses in WM and IgM-MGUS patients, which is the main reason why molecular data are not yet included in the diagnostic criteria. Lastly, studies in patients with familial WM are very limited. Potential contributors to genetic predisposition have been identified and represent possible candidates for validation within different familial WM populations. Family history information should therefore be routinely collected.

In terms of future research directions, since the temporal acquisition of genomic mutations remains unclear, longitudinal studies are needed to explore the dynamics of clonal architecture and to identify driver mutations that play a role in disease course or chemoresistance. Moreover, deciphering the mechanisms of progression in premalignant IgM gammopathies will help to monitor patients at risk of progression. The use of circulating nucleic acids as minimally invasive, disease-specific and dynamic biomarkers is highly promising, but further research is needed to develop signatures with high specificity and sensitivity that can be routinely implemented in clinical practice. In the same regard, an interesting future perspective is combining cell-free DNA and RNA, as well as other cellular molecules, in a multi-omics approach. Lastly, in contrast to different solid and hematological tumors, the role of (epi)miRNAs interaction, lncRNAs and circRNAs have been very sparsely investigated in WM and IgM-MGUS. Including these dimensions could significantly contribute to our understanding of these diseases and ultimately to the development of new biomarkers and therapies.

## Figures and Tables

**Table 1:** *MYD88^L265P^* detection in WM and IgM-MGUS. pts: patients; WM: Waldenström macroglobulinemia; MGUS: monoclonal gammopathy of undetermined significance; WGS: whole-genome sequencing; BM CD19+: bone marrow CD19+ selected cells; AS-PCR: allele-specific polymerase chain reaction; AS-qPCR: allele-specific quantitative PCR; FFPE: formalin-fixed paraffin-embedded; WBC: white blood cells; MNC: mononuclear cell; LN: lymph-node; RFLP: restriction fragment length polymorphism; PB: peripheral blood; WES: whole-exome sequencing; LNA: locked nucleic acid; MEMO-PCR: mutant enrichment with 3′ modified oligonucleotides PCR; MPS: massively parallel sequencing; ARMS qPCR: allele refractory mutation system qPCR; PC: plasma cells; dPCR: digital PCR; ND: not described. ES: effect size measured by random-effects meta-analysis. Diagnostic criteria: see Figure 1.

Reference	Technique	Tissue	WM		IgM-MGUS		Diagnostic Criteria
			pts	*MYD88^L265P^*	pts	*MYD88^L265P^*	
**Treon et al., 2012**	WGS Sanger	BM CD19+	30	91%	21	10%	2° IWWM
[27]							
**Landgren et al., 2012**	Sanger	BM CD19+			9	56%7	2° IWWM
[38]							
**Gachard et al., 2013**	PCR	BM	27	67%			WHO 2008
[39]							
**Xu et al., 2013**	SYBR AS-qPCR	BM CD19+	104	93%	24	54%	2° IWWM
[40]							
**Ondrejka et al., 2013**	AS-PCR	BM biopsy FFPE	13	100%			WHO 2008
[41]							
**Jimenez et al., 2013**	AS-qPCR	BM/PB WBC	117	86%	31	87%	WHO 2011
[42]							
**Poulain et al., 2013**	PCR	BM CD19+	67	79%			2° IWWM
[43]							
**Willenbacher et al., 2013**	Sanger	BM biopsy FFPE	7	86%			2° IWWM
[44]							
**Mori et al., 2013**	AS-PCR Sanger	BM MNC	25	76%			2° IWWM
[45]							
**Varettoni et al., 2013**	AS-PCR	BM MNC	58	100%	77	47%	2° IWWM
[46]							
**Argentou et al., 2014**	PCR-RFLP	BM-PB WBC,	12	92%	1	100%	WHO 2008
[47]		BM CD19+					
**Capaldi et al., 2014**	AS-PCR	BM biopsy FFPE	32	97%	21	43%	ND
[48]							
**Petrikkos et al., 2014**	AS-PCR	BM biopsy-MNC-slides	29	66%			2° IWWM
[49]							
**Ansell et al., 2014**	WES, Sanger AS-qPCR	LN-BM biopsy PC	39	97%			ND
[50]							
**Hunter et al., 2014**	WGS	BM CD19+	30	90%			2° IWWM
[28]							
**Xu et al., 2014**	AS-qPCR	BM-PB CD19+	118	97%	12	42%	2° IWWM
[51]							
**Treon et al., 2014**	AS-PCR	BM CD19+	175	90%			2° IWWM
[52]							
**Patkar et al., 2015**	AS-PCR	BM slides	32	84%			WHO 2008
[53]							
**Schmidt et al., 2015**	LNA-clamped PCR	BM biopsy FFPE	51	96%			2° IWWM WHO 2008
[54]							
**Shin et al., 2016**	MEMO-PCR	BM slides	28	75%			ND
[55]							
**Burnworth et al., 2016**	PCR	BM C19+	21	100%			WHO 2008
[56]		PC					
**Correa et al., 2017**	ARMS qPCR	BM biopsy FFPE	42	82%	55	27%	mSMART
[57]							
**Varettoni et al., 2017**	RT-qPCR	BM CD19+	130	86%	130	60%	2° IWWM
[10]	MPS		62	85%	57	47%	
**Baer et al., 2017**	AS-qPCR	BM/PB MNC	78	86%			ND
[58]	MPS		78	69%			
**Paludo et al., 2017**	ARMS AS-PCR	BM	29	86%			2° IWWM
[59]							
**Cao et al., 2017**	AS-qPCR Sanger	BM CD19+	42	93%	18	44%	2° IWWM
[60]							
**Abeykoon et al., 2018**	AS-PCR	BM	219	79%			mSMART
[61]							
**Drandi et al., 2018**	dPCR	BM/PB WBC	133	96%	4	100%	WHO 2011
[62]							
**Vinarkar et al., 2019**	AS-PCR Sanger	BM/PB—BM slides	33	85%			WHO 2008
[63]							
**Nakamura et al., 2019**	MPS	PB MNC	19	74%	21	67%	WHO 2008
[64]							
**Wu et al., 2020**	AS-qPCR	BM/PB MNC	27	89%			2° IWWM
[65]							
**Wang et al., 2021**	MPS	BM	68	84%			2° IWWM
[66]							
**Kofides et al., 2021**	AS-PCR	BM	391	96%			2° IWWM
[67]	MPS			66%			

**WM:** ES (95% CI) = 0.88 (0.87–0.90). Heterogeneity: *Q*-value = 211, df = 33 (*p* = 0.000), I^2^ = 84.4%. **IgM-MGUS:** ES (95% CI) = 0.54 (0.40–0.67). Heterogeneity: *Q*-value = 96, df = 12 (*p* = 0.000), I^2^ = 87.5%.

**Table 2:** *CXCR4* detection in WM and IgM-MGUS. pts: patients; WM: Waldenström macroglobulinemia; MGUS: monoclonal gammopathy of undetermined significance; BM CD19+: bone marrow CD19+ selected cells; FFPE: formalin-fixed paraffin-embedded; AS-PCR: allele-specific polymerase chain reaction; AS-qPCR: allele-specific quantitative PCR; MPS: massively parallel sequencing; MNC: mononuclear cell; BM: bone marrow; PC: plasma cells; PB: peripheral blood; ES: effect size measured by random-effects meta-analysis.

Reference	Technique	Tissue	WM		IgM-MGUS		Diagnostic Criteria
			pts	*CXCR4^MUT^*	pts	*CXCR4^MUT^*	
**Treon et al., 2014** [52]	Sanger	BM CD19+	175	29%			2° IWWM
**Roccaro et al., 2014** [90]	AS-qPCR	BM CD19+	131	28%	40	20%	WHO 2011
**Hunter et al., 2014** [28]	WGS Sanger	BM CD19+	177	29%			2° IWWM
**Schmidt et al., 2015** [54]	Sanger	BM biopsy FFPE	47	36%			2° IWWM WHO 2008
**Xu et al., 2016** [87]	AS-PCR Sanger	BM CD19+	164	40%	12	17%	2° IWWM
**Poulain et al., 2016** [84]	MPS Sanger	BM CD19+	98	25%			2° IWWM
**Burnworth et al., 2016** [56]	PCR	BM CD19+ PC	27	47%			WHO 2008
**Cao et al., 2017** [60]	Sanger AS-qPCR	BM CD19+	42	24%	18	6%	2° IWWM
**Varettoni et al., 2017** [10]	Sanger	BM CD19+	130	22%	130	4%	2° IWWM
	MPS		62	23%	57	9%	
**Baer et al., 2017** [58]	MPS	BM/PB MNC	69	25%			ND
**Guerrera et al., 2018** [83]	AS-PCR Sanger	BM CD19+	33	66%			2° IWWM
**Vinarkar et al., 2019** [63]	Sanger	BM/PB or BM slides	28	7%			WHO 2008
**Castillo et al., 2019** [85]	AS-PCR Sanger	BM CD19+	180	38%			2° IWWM
**Wu et al., 2020** [65]	AS-qPCR	BM/PB MNC	27	4%			2° IWWM
**Wang et al., 2021** [66]	AS-qPCR	BM	68	37%			2° IWWM
**Gustine et al., 2021** [92]	AS-PCR, Sanger	BM CD19+	107	40%			2° IWWM
	MPS	BM	107	15%			

**WM:** ES (95% CI) = 0.29 (0.23–0.34). Heterogeneity: *Q*-value = 103.68, df = 17 (*p* = 0.000), I^2^ = 83.6%. **IgM-MGUS:** ES (95% CI) = 0.084 (0.027–0.140). Heterogeneity: *Q*-value = 6.73, df = 4 (*p* = 0.151), I^2^ = 40.6%.

**Table 3:** Infrequent DNA mutations in WM and IgM-MGUS. pts: patients; MUTs: mutations; WM: Waldenström macroglobulinemia; MGUS: monoclonal gammopathy of undetermined significance; WGS: whole-genome sequencing; MPS: next-generation sequencing.

Gene	Technique	WM		IgM-MGUS		Reference
		pts	MUTs	pts	MUTs	
**KMT2D**	WGS	18	22%			**Hunter et al., 2018** [82]
	MPS	62	24%	57	5%	**Varettoni et al., 2017** [10]
**TP53**	WGS	30	7%			**Hunter et al., 2014** [28]
	MPS	125	7%	10	0%	**Poulain et al., 2017** [111]
	MPS	62	10%	57	5%	**Varettoni et al., 2017** [10] **Wang et al., 2021** [66]
	MPS	68	12%			
**ARID1A**	WGS, Sanger	30	17%			**Treon et al., 2012** [27]
	WGS	30	17%			**Hunter et al., 2014** [28]
	MPS	62	5%	57	2%	**Varettoni et al., 2017** [10]
	WGS, targeted MPS	85	8%			**Roos-Weil et al., 2019** [112]
**CD79B**	WGS	30	7%			**Hunter et al., 2014** [28]
	MPS	98	12%			**Poulain et al., 2016** [84]
	MPS	62	3%	57	2%	**Varettoni et al., 2017** [10]
**MYBBP1A**	WGS	30	7%			**Hunter et al., 2014** [28]
**NOTCH2**	WGS	30	3%			**Hunter et al., 2014** [28]
	MPS	62	5%	57	9%	**Varettoni et al., 2017** [10]
**PRDM1**	MPS	62	6%	57	2%	**Varettoni et al., 2017** [10]
**TRAF3**	WGS	30	3%			**Hunter et al., 2014** [28]
	MPS	62	2%			**Varettoni et al., 2017** [10]
**SPI1**	WGS, targeted MPS	85	6%			**Roos-Weil et al., 2019** [112]
	TWIST custom capture	239	4%			**Krzisch et al., 2021** [110]

**Table 4:** mRNA expression in WM. DE: differentially expressed; HC: healthy control; MM: multiple myeloma; MM-PC: MM’s plasma cell; PB: peripheral blood; RT-qPCR: reverse transcription-quantitative PCR; WM: Waldenström macroglobulinemia; WM-BL: Waldenström’s B lymphocyte; WM-PC: Waldenström’s plasma cell.

Reference	Method	Sample	RNA	Level	Result
**Chng et al. 2006** [124]	microarray	BM: 23 WM (CD19+/ CD138+); 101 MM (CD138+); 24 SMM (CD138+); 22 MGUS (1 IgM-MGUS: CD19+/CD138+); 15 NPC (CD138+) PB: 7 NBL (CD19+)/8 CLL (CD19+)	48 mRNA (top 10: IL6, NRGN, P311, OSBPL3, CD1C, GPR30, HSU54999, GPR30, SLC2A3, TIP-1, WHSC1)	up	upregulated in WM compared to CLL/MM
			25 mRNA (top 10: DKFZP564A2416, KLF13, WBSCR14, PDE1C, CLDN1, DD96, CHRNA4, CST4, LY9, OPRK1)	down	downregulated in WM compared to CLL/MM
**Gutiérrez et al, 2007** [128]	microarray	BM: 10 WM BL/PC (combination of CD10/CD19/CD38/CD34/CD45/K-L); 12 MM, 11 CLL (CD19+/CD5+); 5 NPC (CD38+) PB: 8 NBL (CD19+)	ABCB4, IL4R, ADAM28, ITPR1, SESN1, BACH2, ABCB1, ADARB1, APLP2, GABBR1	down	downregulated in WM-BL compared to CLL/ NBL
			IL6, NR4A2, HCK, DUSP1, EBI2, FAM46C, TNFRSF13B, FOSB, S100A8	up	upregulated in WM-BL compared to CLL/NBL
			IGLV2-14, DEK, HLA-DMA, HMGB1, CPA3, MS4A3, MYB, HLA-DPA1, RNASE2, CLC, EBI2, SYK, HLA-DRB1	up	upregulated in WM-PC compared to MM-PC/NPC
			LEF1, ATXN1 and FMOD (down), MARCKS (up)		this signature discriminated between clonal WM-BL and CLL
**Hunter et al, 2010** [132]	microarray RT-qPCR	BM: 40 WM/15 normal B cells (CD19+)	IRS2, PIK3R1	down	downregulated in WM compared to NBL
**Roccaro et al, 2010** [133]	microarray	BM:6 WM (CD19+) PB: 2 NBL (CD19+)	HDAC-2, -4, -5, -6, -8, and -9	up	upregulated in primary WM-BL
			HAT-1, -2, and -3	down	downregulated in primary WM-BL
**Sun et al, 2011** [134]	microarray RT-qPCR	BM: 30 treated WM-BL (CD19+) PB:C 10 HC (CD19+) BM:5 treated WM (CD19+) PB: 5 HC (CD19+)	HDAC4, HDAC9, Sirt5	up	upregulated in WM compared to NBL
			HDAC9	up	upregulated in WM compared to NBL, no differential expression for HDAC4 and Sirt5 in RT-qPCR validation
**Gaudette et al, 2016** [130]	microarray	10 WM-BL/PC; 11 CLL; 12 MM; 8 NBL; 5 NPC	BAK1, BCL2L11, MCL1, BCL2L2	down	downregulated in WM-PC compared to MM
			BID	up	upregulated in WM-PC compared to MM
			BID, BCL2A1	up	upregulated in WM-BL compared to CLL
			BAK1	down	downregulated in WM-BL compared to CLL
			BAX, BCL2A1, BBC3, BCL2, NOXA	up	upregulated in WM-BL compared to NBL
**Hunter et al, 2016** [104]	RNA-seq	BM: 57 WM-BL (CD19+) PB: normal nonmemory B-cells (CD19+/CD27-)/memory B-cells (CD19+/CD27+)	DNTT, RAG1, RAG2, IGF1, BMP3, CD5L, CXCL12, VCAM1, CXCR4, B2M, BCL2, BCL2L1 CXCR4, CD79A, CD79B (among 13 571 DE genes)	up	upregulated in WM-BL compared to NBL
			BAX (among 13 571 DE genes)	down	downregulated in WM-BL compared to NBL
			*IL6, IRAK2, TNFAIP3, NFKBIZ, NFKB2, TIRAP,* *PIM1, PIM2, CD40* (among 1155 DE genes)	up	upregulated in *MYD88^L265P^* WM-BL compared to *MYD88^WT^* WM-BL
			PTBP3, CD86, CXCR3, IGF1R, PIK3AP1, AKT2 among 1155 DE genes	down	downregulated in *MYD88^L265P^* WM-BL compared to *MYD88^W^*^T^ WM-BL
			TLR4, IL15, WNT5A, PRDM5, CXXC4, CKDN1C, WNK2, CABLES1, IL17RB, GPER1, IGF1, PMAIP1, RGS1, RGS2, RGS13, DUSP1, DUSP2, DUSP4, DUSP5, DUSP10, DUSP16, DUSP22, ERRFI1 (among others)	down	downregulated in *MYD88^L265P^/CXCR4^WHIM^* WM-BL versus *MYD88^L265P^/CXCR4^WT^* WM-BL
			IRAK3, CXCR7, TLR7, TSPAN33, PIK3R5, PIK3CG (among others)	up	upregulated in *MYD88^L265P^/CXCR4^WHIM^* WM-BL versus *MYD88^L265P^/CXCR4^WT^* WM-BL
			HIVEP2, BCLAF1, FOXO3, ARID1B (among 131 DE genes)	down	downregulated in WM-BL with 6q deletions

**Table 5:** miRNA expression in WM. AFM: atomic force microscopy; BL: B lymphocyte; BM: bone marrow; CLL: chronic lymphatic leukemia; DC: differential centrifugation; HC: healthy control; MGUS: monoclonal gammopathy of undetermined significance; MM: multiple myeloma; NBL: normal B lymphocyte; NPC: normal plasma cell; PB: peripheral blood; PC: plasma cell; QC: quality control; RT-qPCR: reverse transcription-quantitative PCR; SMM: smoldering multiple myeloma; TEM: transmission electron microscope; WM: Waldenström macroglobulinemia.

Reference	Method	EV Purification (QC)	Sample	RNA	Level	Result
***Diagnosis***						
**Roccaro et al, 2009** [135]	liquid phase Luminex microbead miRNA profiling RT-qPCR	NA	BM: 15 R/R WM (CD19+); 5 untreated WM (CD19+), 3 NBL (CD19+) PB: 3 NBL (CD19+)	miR-363-5p, miR-206, miR-494, miR-155, miR-184, miR-542-3p	up	upregulated in WM compared to NBL
				miR-9-3p	down	downregulated in WM compared to NBL
**Hunter et al, 2010** [132]	microaray RT-qPCR	NA	BM: 11 WM (CD19+); 5 NBL (CD19+)	miR-21, miR-29c, miR-155	up	upregulated in WM compared to NBL
				miR-9-3p, miR-27b, miR-126-3p, miR-126-5p, miR-145, miR-223, miR-886-5p	down	downregulated in WM compared to NBL
**Roccaro et al, 2010** [133]	liquid-phase Luminex microbead miRNA profiling RT-qPCR	NA	BM:6 WM (CD19+) PB: 2 NBL (CD19+) BCWM.1 cell line	miR-206-3p	up	upregulated in WM-BL compared to NBL
				miR-9-3p	down	downregulated in WM-BL compared to NBL
**Fulciniti et al, 2016** [136]	microarray	NA	BM: WM (CD19+) PB: NBL (CD19+)	miR-23b	down	downregulated in WM compared to NBL
**Caivano et al, 2017** [137]	RT-qPCR	DC (AFM/TEM)	PB:14 WM;18 HC	miR-155	up	upregulated in WM compared to HC
**Gaudette et al, 2016** [130]	RT-qPCR	NA	BCWM.1, MWCL-1, RPCI-WM11 cell lines	miR-155-5p	up	upregulated in BCWM.1 and MWCL-1 cells but not RPCI-WM1 cells
**Bouyssou et al, 2018** [138]	microarray	DC (TEM/particle size analysis)	BM: 6 WM (CD19+) PB: 30 smouldering WM; 44 symptomatic WM; 10 HC	miR-192-5p, miR-93-5p, miR-15a-5p, miR-16-5p, miR-20a-5p, miR-378a-3p	up	upregulated in smouldering WM compared to HC
				miR-199a-5p, miR-145-5p, miR199a-3p, miR-221-3p, miR-335-5p, let-7d-5p	down	downregulated in smouldering WM compared to HC
**Hodge et al, 2011** [139]	microarray	NA	BM/PB: 8 WM (CD19+/CD138+); 6 WM-BL (CD19+); 3 WM-PC (CD138+), 5 MM (CD138+); 5 CLL (CD19+); 3 NBL (CD19+); 6 NPC (CD138+); 4 normal CD19+/CD138+ B-cells	miR-193b-3p, miR-126-3p, miR-181a-5p, miR-125b-5p, miR-451a	up	upregulated in combined WM (CD19+, CD 138+, CD19+/CD138+) vs CLL
				miR-92a-3p, miR-223-3p, miR-92b-3p, miR-363-3p	up	upregulated in combined WM vs MM
				miR-9-3p, miR-193b-3p, miR-182-5p, miR-152-3p	down	downregulated in combined WM vs MM
				miR-21-5p, miR-142-3p	up	upregulated in combined WM (CD19+, CD 138+, CD19+/CD138+) vs NBL
				miR-182-5p, miR-152-3p, miR-373-5p, miR-575-3p	down	downregulated in combined WM (CD19+, CD 138+, CD19+/CD138+) vs NBL
**Kubiczkova et al, 2015** [140]	Microarray RT-qPCR	ExoQuick	PB: 21 WM (CD19+ and CD19-); 15 igM-MGUS; 10 IgM MM; 18 HC	miR-320a-3p, miR-320b-3p	down	downregulated in WM vs HC vs IgM-MGUS and vs IgM-MM
				miR-151-5p, let-7a-5p	down	downregulated in WM vs. HC and vs. IgM-MGUS
***Therapy Response***						
**Bouyssou et al, 2018** [138]	microarray	DC (TEM/particle size analysis)	PB: 30 smouldering WM; 44 symptomatic WM; 10 HC	miR-21-5p, miR-192-5p, miR-320b-3	up	increased expression with disease progression
				let-7d-5p	down	decreased expression with disease progression
**Roccaro et al, 2012** [141]	RT-qPCR		BM: 4 R/R WM (CD19+) PB: NBL (CD19+) BCWM.1, MEC.1, and RL cell lines	miR-155	NA	everolimus exerts anti-WM activity by targeting miR-155
***Prognosis***						
**Roccaro et al, 2009** [135]	liquid phase Luminex microbead miRNA profiling RT-qPCR	NA	BM: 15 R/R WM (CD19+); 5 untreated WM (CD19+); 3 NBL (CD19+) PB: 3 NBL (CD19+)	miR-363-5p, miR-206, miR-494, miR-155, miR-184, miR-542-3p	up	upregulation is associated with worse IPSS score

**Table 6:** DNA mutations detected in Liquid Biopsy studies in WM and/or IgM-MGUS. AS-PCR: allele-specific polymerase chain reaction; AS-qPCR: allele-specific quantitative PCR; Cast: competitive allele-specific TaqMan PCR; dPCR: digital PCR; ND: not described.

References	Technique	Tissue	WM				IgM-MGUS				Diagnostic Criteria
			Pts	*MYD88^L265P^*	Pts	*CXCR4^MUT^*	Pts	*MYD88^L265P^*	Pts	*CXCR4^MUT^*	
**Bagratuni et al., 2018**	AS-PCR	plasma	79	80%	16	17%	7	86%	9	22%	ND
[189]											
**Drandi et al., 2018**	dPCR	plasma	60	88%							WHO 2011
[62]											
**Wu et al., 2020**	AS-qPCR	plasma	27	85%	27	4%					2° IWWM
[65]											
**Ntanasis-Stathopoulos et al., 2020**	AS-PCR Sanger	plasma	188	89%	131	36%					ND
[191]											
**Ferrante et al., 2021**	dPCR	plasma	32	78%			4	75%			2° IWWM
[99]											
**Demos et al., 2021**	AS-qPCR	plasma	28	68%	23	17%					ND
[190]											
**Bagratuni et al., 2022**	Cast-PCR	plasma	92	88%			51	80%			ND
[192]											

**Table 7:** miRNA detected in Liquid Biopsy studies in WM. AFM: atomic force microscopy; BM: bone marrow; DC: differential centrifugation; HC: healthy control; MGUS: monoclonal gammopathy of undetermined significance; MM: multiple myeloma; PB: peripheral blood; PC: plasma cell; QC: quality control; RT-qPCR: reverse transcription quantitative PCR; TEM: transmission electron microscope; WM: Waldenström macroglobulinemia.

Reference	Method	EV Purification (QC)	Sample	RNA	Level	Result
***Diagnosis***						
**Caivano et al, 2017** [137]	RT-qPCR/ serum	DC (AFM/TEM)	PB:14 WM;18 HC	miR-155	up	upregulated in WM compared to HC
**Bouyssou et al, 2018** [138]	microarray/ plasma	DC (TEM/particle size analysis)	PB: 30 smouldering WD; 44 symptomatic WM; 10 HC	miR-192-5p, miR-93-5p, miR-15a-5p, miR-16-5p, miR-20a-5p, miR-378a-3p	up	upregulated in smouldering WM compared to HC
				miR-199a-5p, miR-145-5p, miR199a-3p, miR-221-3p, miR-335-5p, let-7d-5p	down	downregulated in smouldering WM compared to HC
**Kubiczkova et al, 2015** [140]	microarray RT-qPCR/ serum	ExoQuick	PB: 21 WM (CD19+ and CD19-); 15 IgM-MGUS; 10 IgM-MM; 18 HC	miR-320a-3p, miR-320b-3p	down	downregulated in WM vs HC vs IgM-MGUS and vs IgM-MM
				miR-151-5p, let-7a-5p	down	downregulated in WM vs HC and vs IgM-MGUS
